# An observational study of response heterogeneity in children with attention deficit hyperactivity disorder following treatment switch to modified-release methylphenidate

**DOI:** 10.1186/1471-244X-13-219

**Published:** 2013-09-03

**Authors:** Christopher Hautmann, Aribert Rothenberger, Manfred Döpfner

**Affiliations:** 1 Department of Child and Adolescent Psychiatry and Psychotherapy, University of Cologne, Robert-Koch-Str. 10, 50931 Cologne, Germany; 2 Department of Child and Adolescent Psychiatry, University Medicine Göttingen, von Sieboldstr 5, 37075 Göttingen, Germany

**Keywords:** ADHD, Methylphenidate, Modified-release MPH, Growth mixture modelling, Trajectory

## Abstract

**Background:**

Methylphenidate (MPH) has been shown to be effective in the treatment of attention deficit hyperactivity disorder (ADHD) in children. The overall population of children and adolescents with ADHD may comprise distinct clusters of patients that differ in response to MPH. The aim of this analysis was to look for subgroups with different treatment trajectories and to identify their distinctive features.

**Methods:**

OBSEER was a prospective, observational study examining the effectiveness and safety of once-daily modified-release MPH over 3 months in patients (aged 6–17 years) with ADHD under routine care. Assessments were carried out at baseline (Visit 1), after 1–3 weeks (Visit 2) and 6–12 weeks (Visit 3) after first use of once-daily modified-release MPH. Change in ADHD symptoms, as rated by parents and teachers, was examined post hoc in patients of the intent-to-treat-population (*N* = 822), using growth-mixture modelling to detect response trajectory groups after switching medication. Age, MPH dose at Visit 1 before medication switch, prescribed once-daily modified-release MPH dose at Visits 1 and 2, conduct problems and emotional symptoms were considered predictors of response subgroups.

**Results:**

Assessing formal statistical criteria and usefulness of the models, a 4-class solution best fitted the data: after switching medication two response groups with severe symptoms at study start and subsequent substantial treatment effects, and two showing no or comparatively little treatment effect, one of which had severe and the other less severe symptoms at study start. Patient age, conduct problems and MPH dose at Visit 1 were predictors of inclusion in subgroups.

**Conclusions:**

Older children and children with few conduct problems were more likely to be members of a patient cluster with fewer symptoms at study start. Children with a low MPH dose before medication switch had a higher chance of being in the patient cluster with a strong treatment response after switching medication. The current analyses should assist in identifying children likely to achieve a favourable treatment course with MPH and, additionally, those who are in need of alternative treatment options.

## Background

For children with severe symptoms of attention deficit hyperactivity disorder (ADHD), methylphenidate (MPH) is one of the major treatment options. In randomized placebo-controlled trials, MPH has been shown to be effective and to be associated with, on average, large treatment effects [1,2]. In addition to improving symptom control, MPH can have favourable effects on comorbid oppositional symptoms [3,4] and social functioning [5,6]. Side effects can occur and may require adaptation of the treatment plan [7]. Because of its efficacy and tolerability profile, MPH is recognized in treatment guidelines in Europe and the USA [8-11].

The response achieved by drug treatment varies among children with ADHD; some children will experience adequate symptom control, whereas for others benefits will be minor [12]. There is currently little information on response prediction in ADHD, and detecting and explaining differential treatment effects should be considered an important part of the general treatment research agenda [13,14].

Given the variation in responsiveness, it is reasonable to suggest that the population of patients treated with MPH is heterogeneous and may comprise distinct subgroups, which could be identified. For example, in therapy-naïve children receiving MPH for the first time, two subgroups might be hypothesized to exist: responders, showing good symptom reduction over time, and partial responders, who show few or no treatment effects. Subpopulations can be defined based on ‘observed’ variables that are directly measureable characteristics (e.g., comorbidity). For example, Ghuman et al. [15] found that comorbidities in preschool children with ADHD predicted response to MPH; the subgroup of children with three or more comorbid disorders did not respond to treatment compared with those with two or fewer comorbidities.

Subgroups may not always be identifiable by ‘a priori’-defined measureable characteristics and instead have to be inferred from the data [16,17]. As in this case, group membership is not known beforehand, so these subgroups can be called ‘latent’ or ‘unobserved’ [18]. In clinical research, there is growing interest in the detection of unobserved subgroups associated with differential treatment effects [19]. However, in the treatment of ADHD, knowledge in this area remains very limited. In analyses by Sonuga-Barke et al. [20] of data from COMACS (Comparison of Methylphenidates in the Analog Classroom Setting) [21] and by Swanson et al. [22] of data from the MTA (Multimodal Treatment Study of Children with ADHD) [23], subgroups could be identified and subgroup membership could be linked to differential treatment effects. Owing to differences in methodology and study design, including varying observation periods of 12 hours (COMACS) and 3 years (MTA), direct comparisons of the results of these analyses are not meaningful. Nonetheless, the conclusion from both studies was that, for symptom change in children with ADHD, the overall population is heterogeneous and it is reasonable to look for subgroups with different treatment responses.

MPH is available in immediate-release and extended-release formulations [24]. For immediate-release preparations, the average duration of action is about 4 hours; extended-release formulations are designed to be effective for 8–12 hours [25,26]. Several extended-release formulations, with different compositions and pharmacokinetic properties, are available [27,28]. Meta-analytical comparisons point to similar effect sizes for both delivery forms of MPH [29]. As both immediate- and extended-release formulations have advantages and disadvantages, treatment guidelines often provide recommendations specifying which delivery format is appropriate given a patient’s individual circumstances [27].

Equasym XL® (Shire Pharmaceuticals Ireland Ltd) [30] is a modified-release MPH (MR-MPH) formulation comprising 30% immediate-release coated beads and 70% extended-release coated beads, delivering an initial rapid rise in plasma concentration and a slower rise over about 3 hours, with therapeutic plasma concentrations and efficacy in symptom reduction maintained for about 8 hours. The safety profile and efficacy of MR-MPH have been demonstrated in controlled clinical trials [31].

The OBSEER study (OBservation of Safety and Effectiveness of Equasym XL in Routine care), a 3-month, prospective, observational study involving children and adolescents with ADHD in Germany, was conducted under routine care conditions to gain insights into the effectiveness and safety of treatment with MR-MPH in everyday use. The study included children and adolescents diagnosed with ADHD for whom therapy with MR-MPH was already planned by the attending physician. Patients could have a treatment history with other MPH formulations, other drugs for ADHD or non-pharmacological interventions, or could be treatment naïve. In OBSEER, statistically significant treatment improvements in ADHD symptoms were seen from baseline to last visit [32]. The effectiveness of MR-MPH was rated better than prior therapy by both teachers and parents at all measured time points across the day, particularly late morning and early afternoon.

The aim of the present analysis was twofold. First, to identify subgroups with different treatment response trajectories, thereby extending previous work to attain a more complete picture of response heterogeneity during treatment [20,22]. Second, to identify predictors of subgroups with differential responsiveness that might allow physicians to better select the appropriate treatment for an individual patient.

## Methods

### Participants

Patients aged 6–17 years with a confirmed diagnosis of ADHD according to the *Diagnostic and Statistical Manual of Mental Disorders, 4th Edition, Text Revision* (DSM-IV-TR) [33] or hyperkinetic disorder (HKD) according to the International Classification of Diseases, Version 10 (ICD 10) [34], who were attending school and for whom therapy with MR-MPH (10, 20 or 30 mg once daily) was indicated and already intended by the attending physician, were eligible to participate in the study. Exclusion criteria included mental disability and contraindications for MR-MPH [30].

### Study design

OBSEER was a non-interventional, uncontrolled, multicentre, prospective, observational study conducted in 169 centres in Germany. Written informed consent was obtained from parents. As this was an open observational study under conditions of routine care, there was no IRB-approval necessary according to German rules and European regulations [35,36].

Participating physicians (specialists in paediatrics and/or child and adolescent psychiatry) selected appropriate patients for whom therapy with MR-MPH was indicated. The planned observation period for each patient was 6–12 weeks after the first use of MR-MPH and included three visits: prior to the start of MR-MPH treatment (Visit 1), and 1–3 weeks (Visit 2) and 6–12 weeks (Visit 3) after the first use of MR-MPH in accordance with standard practice. On average, Visit 2 took place 3.5 weeks after Visit 1, and Visit 3 took place 10.5 weeks after the start of the observational period. MR-MPH was prescribed for the first time at Visit 1 and the dose was adjusted, when necessary, at Visits 2 and 3. The study started on 2 November 2006; inclusion of patients concluded on 28 February 2007, and observation was completed on 27 December 2007 [32].

### Assessments

The outcome variable used to describe the response trajectory was the mean symptom score, calculated using the German ADHD Symptom Checklist (*Fremdbeurteilungsbogen für Aufmerksamkeitsdefizit-Hyperaktivitätsstörung* [FBB-ADHD]), which is part of the German Diagnostic System for Mental Disorders in Children and Adolescents (DISYPS) [37]. This checklist assesses diagnostic criteria for ADHD according to DSM-IV-TR and for HKD according to ICD-10. Twenty items are rated on a 4-point scale (0–3), with higher scores indicating more severe symptoms. The overall symptom score (range 0–3) represents the sum of the individual item scores divided by the number of items. Parents and teachers rated items separately at each visit; the analyses included information from both informants.

Several covariates were considered for the prediction of subgroups of responsiveness: patient age; the daily dose (mg) of MPH before switch to MR-MPH (MPH_pre_) as recorded at Visit 1 (for MPH_pre_, patients receiving no medication, or medication other than MPH, were assigned the value 0); the daily dose (mg) of MR-MPH as recorded at Visit 1 (MPH_Visit 1_) and Visit 2 (MPH_Visit 2_); and the patient’s conduct problems (5 items) and emotional symptoms (5 items), as rated by parents at Visit 1 using the Strengths and Difficulties Questionnaire (SDQ) [38] on a scale of 0–2, with higher scores indicating more severe symptoms and with scale scores representing the sum of the individual item scores (range 0–10).

### Statistical analysis

Growth mixture modelling [39,40] was applied to detect subgroups with varying trajectories of change in ADHD symptoms in parent and teacher ratings. For different informants, separate parallel growth processes were conceived and one common categorical latent variable representing subgroups was taken into account. Both growth models had the same periodicity; separate growth rates were taken into account from Visit 1 to Visit 2 and from Visit 2 to Visit 3 [41].

To detect distinctive features of the subgroups, the selected covariates (age, MPH_pre_, MPH_Visit 1_, MPH_Visit 2_, conduct problems, emotional symptoms) were included in the model as predictors (conditional growth mixture model). Regression of a latent categorical variable on the covariates represents a multinomial logistic regression analysis. All covariates were z-transformed before being analysed. To provide an explicit overview of the interrelationships between covariates and subgroups, unstandardized means and standard deviations of covariates in the respective subgroups were calculated: values were obtained by weighting raw data with the estimated posterior probabilities [42,43]. For the conditional growth mixture model, two to seven classes were considered.

The target sample for analysis was the 822 evaluable patients of the intent-to-treat population in OBSEER, as described by Döpfner et al., previously [32]. For growth mixture modelling, missing data were handled using the full-information maximum-likelihood method (FIML) [44,45] and the total sample of the intent-to-treat population was considered. Models with up to seven classes were considered. Model selection was based on a formal statistical criterion, the Bayesian information criterion [BIC] [46], as well as clinical considerations [19]. All analyses were conducted using Mplus software [47].

## Results

### Study population

Evaluable patients in OBSEER (*N* = 822) had a mean (standard deviation) age of 10.04 (2.47) years and 81.25% (663/816) were male. Approximately half of the patients (55.40%; 431/778) had a disturbance of activity and attention (ICD code F90.0), which is similar to ADHD combined type according to DSM-IV; 36.38% (283/778) had a hyperkinetic conduct disorder (F90.1) and 8.23% (64/778) had other hyperkinetic disorders (F90.8). Most patients (69.83%; 574/822) had previously received other MPH formulations; among whom 35.37% (203/574) were previously prescribed extended-release MPH formulations. One quarter of patients (25.30%; 208/822) were treatment naïve; only 4.87% (40/822) received other medications (e.g., atomoxetine, amphetamine) or no medication was specified. Among those previously receiving treatment, the main reasons for switching medication were insufficient overall effectiveness and/or the duration of effect was too short.

### Growth mixture model analysis

Available sample sizes, means and standard deviations for outcome-variable ADHD symptoms and covariates of the growth mixture models are presented in Table 1. A decline in parent-rated and teacher-rated ADHD symptoms was observed in the total study group over the course of the study. The statistical significance of this symptom reduction has already been discussed elsewhere [32]. Mean daily MPH dose was lowest before the switch to MR-MPH and increased from Visit 1 to Visit 2.

**Table 1:** **Available sample sizes, means and standard deviations for ADHD outcome and covariates (*N*** **= 822)**

	***n***	**Mean**	**SD**
*Outcome ADHD*			
Parent rating			
Visit 1	742	1.63	0.62
Visit 2	699	1.16	0.59
Visit 3	637	0.99	0.55
Teacher rating			
Visit 1	570	1.29	0.70
Visit 2	522	1.00	0.61
Visit 3	489	0.85	0.57
*Covariates*			
Age (years)	808	10.04	2.47
MPH_pre_ (mg)	772	16.28	14.77
MPH_Visit 1_ (mg)	802	22.53	9.63
MPH_Visit 2_ (mg)	675	25.25	9.44
Conduct problems^a^	721	4.16	2.29
Emotional symptoms^b^	722	3.79	2.46

ADHD = attention deficit hyperactivity disorder; MPH = methylphenidate; MPH_pre_ = daily dose of MPH before switch to modified-release MPH; MPH_Visit 1_ = daily dose of modified-release MPH prescribed at Visit 1; MPH_Visit 2_ = daily dose of modified-release MPH prescribed at Visit 2; SD = standard deviation.

^a^Conduct Problems Scale Strengths and Difficulties Questionnaire in parent ratings at Visit 1.

^b^Emotional Symptoms Scale Strengths and Difficulties Questionnaire in parent ratings at Visit 1.

**Figure 1:**
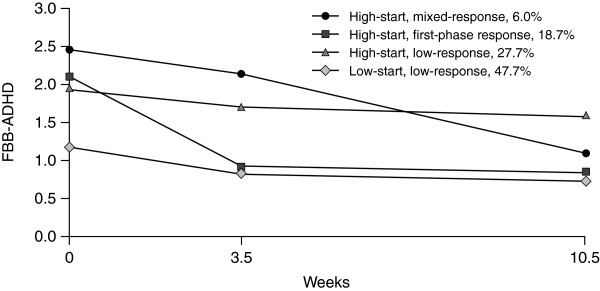
**ADHD symptom trajectories in parent ratings.** ADHD symptom trajectories in parent ratings from Visit 1 to Visit 3 in the four-class solution of the conditional growth mixture model (*N* = 822); time corresponds to the average time the visits took place. ADHD = attention deficit hyperactivity disorder; FBB-ADHD = German ADHD Symptom Checklist (Fremdbeurteilungsbogen für Aufmerksamkeitsdefizit-Hyperaktivitätsstörung). High-start = subgroups with more severe symptoms compared with the low-start group at Visit 1; mixed-response = in parent ratings, comparatively stronger treatment response is observed during Visit 2 to Visit 3 and in teacher ratings during Visit 1 to Visit 2; first-phase response = in parent and in teacher ratings comparatively stronger treatment response is observed during Visit 1 to Visit 2; low-response = compared with other subgroups, only minor symptom reduction is detected during the observational period.

**Figure 2:**
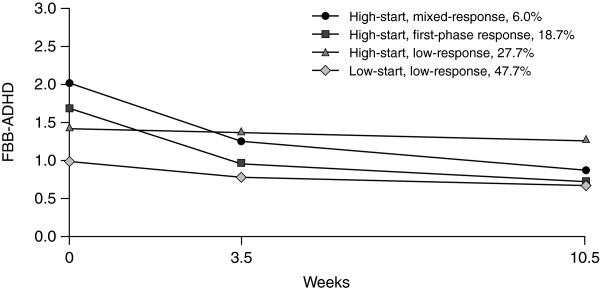
**ADHD symptom trajectories in teacher ratings.** ADHD symptom trajectories in teacher ratings from Visit 1 to Visit 3 in the four-class solution of the conditional growth mixture model (*N* = 822); time corresponds to the average time the visits took place. ADHD = attention deficit hyperactivity disorder; FBB-ADHD = German ADHD Symptom Checklist (Fremdbeurteilungsbogen für Aufmerksamkeitsdefizit-Hyperaktivitätsstörung). High-start = subgroups with more severe symptoms compared with the low-start group at Visit 1; mixed-response = in parent ratings, comparatively stronger treatment response is observed during Visit 2 to Visit 3 and in teacher ratings during Visit 1 to Visit 2; first-phase response = in parent and in teacher ratings comparatively stronger treatment response is observed during Visit 1 to Visit 2; low-response = compared with other subgroups only minor symptom reduction is detected during the observational period.

All conditional growth mixture models converged; BIC values from the two-class solution through to the seven-class solution were 17,201, 17,104, 17,075, 17,064, 17,068 and 17,067, respectively. According to the BIC, the best fit was the five-class solution, but closer inspection revealed a subgroup containing only about 3% of the sample. Assessing formal statistical criteria, and the parsimoniousness and usefulness of the models, the four-class solution was considered to be optimal; subgroup trajectories for this solution are presented for parent (Figure 1) and teacher (Figure 2) ratings. The subgroups identified included one with low symptom scores at Visit 1 (low-start group) and three with higher initial symptom scores (high-start groups). Additionally, subgroups were also divided into those in which symptoms decreased between Visits 1 and 2 with little or no change between Visits 2 and 3 in either parent or teacher ratings (first-phase response); those in which symptoms decreased between Visits 1 and 2 for the parent rating scales and between Visits 2 and 3 for teacher scales (mixed-response); and those in which there were minor changes in symptom scores during the observation period (low-response).

The strongest treatment effects were observed in two of the high-start groups. In the first group (high-start, first-phase response), which included 18.7% (*n* = 154) of all patients, both parents and teachers reported a symptom decrease from Visit 1 to Visit 2, with no or little change from Visit 2 to Visit 3. In the second, smaller, high-start group (high-start, mixed-response), which included only 6.0% (*n* = 49) of all patients, both informants reported strong symptom reduction, but the time during which the change was perceived differed, with the greatest improvement perceived from Visit 1 to Visit 2 for teachers and from Visit 2 to Visit 3 for parents. In the third high-start group (high-start, low-response), which included 27.7% (*n* = 227) of all patients, no or only a modest change was observed in both parent and teacher ratings. Similarly, for the low-start, low-response group, which was the largest subgroup with 47.7% (*n* = 392) of all patients, only minor changes in symptom scores were seen during the observation period.

In comparison with the total sample, children from the high-start, mixed-response and high-start, first-phase response groups could be described as younger, receiving a lower dose of MPH before study start, receiving a lower dose of MR-MPH at Visits 1 and 2, and showing more severe conduct problems and an average level of emotional symptoms (Table 2). Similarly, children in the high-start, low-response group could be characterized as being of average age, receiving an average or slightly above-average dose of MPH before study start, receiving an average dose of MR-MPH at Visits 1 and 2, and demonstrating higher than average conduct problems and pronounced emotional symptoms (Table 2). Children in the low-start group were older, were prescribed average MPH doses before study start, received average doses of MR-MPH at Visits 1 and 2, and exhibited minor conduct problems and an average level of emotional symptoms (Table 2).

**Table 2:** Mean (SD) covariates: total sample and subgroups of the conditional growth mixture model four-class solution

			**Subgroup**							
	**Total sample (*N*** **= 822)**		**High-start, mixed-response (*n*** **= 49)**		**High-start, first-phase response (*n*** **= 154)**		**High-start, low-response (*n*** **= 227)**		**Low-start, low-response (*n*** **= 392)**	
	**Mean**	**(SD)**	**Mean**	**(SD)**	**Mean**	**(SD)**	**Mean**	**(SD)**	**Mean**	**(SD)**
Age (years)	10.04	(2.47)	8.83	(2.33)	9.26	(2.29)	9.96	(2.51)	10.55	(2.38)
MPH_pre_ (mg)	16.45	(14.80)	6.27	(10.39)	9.57	(12.29)	19.42	(15.03)	18.39	(14.61)
MPH_Visit 1_ (mg)	22.51	(9.61)	17.41	(7.81)	19.82	(7.97)	23.00	(10.61)	24.00	(9.38)
MPH_Visit 2_ (mg)	25.52	(9.81)	21.61	(7.47)	23.26	(7.89)	26.10	(11.18)	26.03	(8.90)
Conduct problems^a^	4.16	(2.29)	6.67	(1.84)	5.43	(1.90)	5.17	(2.04)	2.77	(1.67)
Emotional symptoms^b^	3.79	(2.46)	3.78	(2.47)	3.55	(2.51)	4.48	(2.38)	3.48	(2.41)

High-start = subgroups with more severe symptoms compared with the low-start group at Visit 1; mixed-response = in parent ratings, comparatively stronger treatment response is observed during Visit 2 to Visit 3 and in teacher ratings during Visit 1 to Visit 2; first-phase response = in parent and in teacher ratings comparatively stronger treatment response is observed during Visit 1 to Visit 2; low-response = compared with other subgroups only minor symptom reduction is detected during the observational period; Missing data were handled by FIML; to obtain means and SDs in the subgroups, raw data were weighted by the estimated posterior probabilities.

FIML = full-information maximum-likelihood method; MPH = methylphenidate; MPH_pre_ = daily dose of MPH before switch to modified-release MPH; MPH_Visit 1_ = daily dose of modified-release MPH prescribed at Visit 1; MPH_Visit 2_ = daily dose of modified-release MPH prescribed at Visit 2; SD = standard deviation.

^a^Conduct Problems Scale Strengths and Difficulties Questionnaire in parent ratings at Visit 1.

^b^Emotional Symptoms Scale Strengths and Difficulties Questionnaire in parent ratings at Visit 1.

All possible comparisons among subgroups are reported, using the multinomial logistic regression analysis, which provides a statistical test for the significance of a particular covariate while controlling for all other predictor variables (Table 3). Age could be used to discriminate the low-start subgroup from all other subgroups: as age increased, so did the probability of inclusion in the low-start subgroup. MPH dose at pretreatment (MPH_pre_) could discriminate between the two subgroups with good treatment response (high-start, mixed-response; high-start, first-phase response) and the two subgroups with little or no treatment response after switching medication (high-start, low-response; low-start, low-response), with higher pretreatment doses increasing the likelihood of falling into one of the less responsive subgroups. The prescribed dose of MR-MPH at Visit 1 (MPH_Visit 1_) and Visit 2 (MPH_Visit 2_), and emotional symptom scores were not useful for subgroup prediction in either class comparison. Conduct problems, however, showed predictive power, with greater conduct problems reducing the likelihood of inclusion in the low-start, low-response subgroup. Among the three subgroups with higher ADHD symptom scores at study start, higher conduct problem scores increased the likelihood of inclusion in the high-start, mixed-response subgroup.

**Table 3:** Results of multinomial logistic regression with latent class as criterion

	**High-start, mixed response vs. low-start, low response**		**High-start, first-phase response vs. low-start, low response**		**High-start, low-response vs. low-start, low response**		**High-start, mixed response vs. high-start, low response**		**High-start, first-phase response vs. high-start, low response**		**High-start, mixed response vs. high-start, first-phase response**	
	**β**	**OR**	**β**	**OR**	**β**	**OR**	**β**	**OR**	**β**	**OR**	**β**	**OR**
Age (years)	–0.85^a^	0.43	–0.65^a^	0.52	–0.39^a^	0.68	–0.46	0.63	–0.26	0.77	–0.20	0.82
MPH_pre_ (mg)	–1.18^a^	0.31	–0.73^a^	0.48	0.11	1.12	–1.29^a^	0.28	–0.84^a^	0.43	–0.46	0.63
MPH_Visit 1_ (mg)	–0.13	0.88	0.03	1.03	–0.15	0.87	0.02	1.02	0.18	1.19	–0.16	0.85
MPH_Visit 2_ (mg)	–0.07	0.94	–0.02	0.98	0.06	1.06	–0.12	0.88	–0.08	0.92	–0.04	0.96
Conduct problems^b^	2.80^a^	16.40	1.93^a^	6.92	1.60^a^	4.97	1.19^a^	3.29	0.33	1.39	0.87^a^	2.38
Emotional symptoms^c^	–0.31	0.73	–0.26	0.77	0.17	1.18	–0.48	0.62	–0.42	0.66	–0.06	0.95

High-start = subgroups with more severe symptoms compared with the low-start group at Visit 1; mixed-response = in parent ratings, comparatively stronger treatment response is observed during Visit 2 to Visit 3 and in teacher rating during Visit 1 to Visit 2; first-phase response = in parent and in teacher ratings comparatively stronger treatment response is observed during Visit 1 to Visit 2; low-response = compared with other subgroups only minor symptom reduction is detected during the observational period; for analyses, all covariates were z-transformed.

β = regression coefficient; OR = odds ratio; MPH = methylphenidate; MPH_pre_ = daily dose of MPH before switch to modified-release MPH; MPH_Visit 1_ = daily dose of modified-release MPH prescribed at Visit 1; MPH_Visit 2_ = daily dose of modified-release MPH prescribed at Visit 2.

^a^α < 0.05.

^b^Conduct Problems Scale Strengths and Difficulties Questionnaire in parent ratings at Visit 1.

^c^Emotional Symptoms Scale Strengths and Difficulties Questionnaire in parent ratings at Visit 1.

## Discussion

Treatment response to MPH is variable, supporting the contention that the population of children receiving treatment for ADHD is not homogeneous. This analysis sought to identify and determine the distinctive features of clusters of patients with different response trajectories to treatment with MR-MPH in parent and teacher ratings under real world conditions using data from the OBSEER study [32].

After switching medication, four response subgroups were detected: two with substantial treatment effects, both of which had more severe symptoms at study start, and two with little or no treatment effects, one of which had low initial symptom scores and one that had more severe symptoms at study start.

The subgroup with low initial symptom scores was the largest, comprising about half of all children. Here, only small treatment effects were observed, and for adequate interpretation of this finding, the nature of observational studies has to be considered. These children may already have reached good symptom control with the previous medication, leaving relatively little room for symptom improvement with transition to MR-MPH. In this study, the meaning of the term treatment response is therefore to understand the response following the change in treatment relative to previous medication effects and is no indication for absolute efficacy of MR-MPH. However, some symptom reduction was observed in this group. On a descriptive level, children in the low-start subgroup were older and had fewer conduct problems. Statistical tests confirmed that age and conduct problems were useful predictors to distinguish the low-start subgroup from the other groups, which concurs with previous research findings. Generally, it is accepted that ADHD symptoms decrease with age, although the finding of symptom reduction with age may reflect the developmental insensitivity of the DSM-IV, not the natural history of ADHD [48], and that children without comorbid conduct problems generally exhibit fewer ADHD symptoms than their counterparts with such comorbidities [49].

Approximately one quarter of the children fell into one of two subgroups with a strong response to MR-MPH. One feature distinguishing the two more responsive subgroups from the two more stable subgroups was the MPH dose at study start. On a descriptive level, children in the more-responsive groups were treated less intensively prior to study start; statistical analysis confirmed that lower MPH dose before medication switch was linked to a higher probability of falling into a more-responsive subgroup. Due to the fact that the premedication in the more-responsive subgroups was lower than that in the less-responsive subgroups, the relative increase in MPH dosage in these groups was the highest after the medication switch. These findings are in line with the assumption of a linear dose–response relationship between MPH treatment and ADHD symptoms, as has previously been demonstrated [50,51]. However, more-responsive versus low-response subgroups could not be distinguished by the prescribed MR-MPH doses during the observation period.

One third of children were classified as ‘high-start, low-response’, with higher initial symptom scores and no or only modest treatment effects after switch to MR-MPH. These children represent a population that is difficult to treat. The average MPH dose was about 19 mg/day at study start and 26 mg/day at Visit 2 for this subgroup. For patients in this cluster, adjustment or change of medication or additional psychosocial interventions should be considered [52,53].

Emotional symptoms were not useful for distinguishing between subgroups. Early work addressing the importance of anxiety on the effect of stimulant medication in ADHD indicated that behavioural effects are weaker in anxious children [54]. From this conclusion, for the current analysis, it would have been expected that more anxious children would be less likely to be in one of the more-responsive groups. However, more recent studies have not replicated these findings [55,56], and Tannock [57] suggests that current evidence points to similar effects of stimulant medication in children with and without comorbid anxiety. The current analysis is in line with this evaluation.

Conduct problems proved to be useful for distinguishing subgroups with more-severe ADHD symptoms from the subgroup with less-severe symptoms at study start, but this variable was less suited to differentiating between subgroups with and without a good treatment response after switching medication. Connor et al. [3] suggest that the effects of pharmacotherapy are similar in children with ADHD, both with and without comorbid conduct problems, which corresponds with our findings.

In OBSEER, Döpfner et al. [58] reported that treatment-naïve as well as pretreated children demonstrated ADHD symptom reduction during the observational period of the study, and that the greatest reduction was seen in treatment-naïve patients. In the present analysis, these two groups were not explicitly distinguished, but it was found that less-intensively pretreated children were more likely to be members of one of the two response subgroups, which is in line with the previous analysis.

The advancement of evidence-based medicine has had a major impact on the healthcare system and intervention research [59,60]. High quality studies are required to demonstrate the efficacy of a particular treatment [61]. The quality of evidence from clinical studies varies depending on the study design and, in general, evidence from randomized, controlled trials is considered to be of high quality and that from observational studies is sometimes considered to be of low quality [62]. Nevertheless, both research designs have strengths and limitations, and instead of regarding them in a strictly hierarchical fashion, with randomized, controlled trials as the gold standard, a different perspective is to view them as complementary research designs [63,64]. Randomized, controlled trials are high on internal validity but there may be a lack of generalizability of their findings. For example, in the MTA, the largest clinical trial in ADHD to date, children with irregular school visits were not eligible for participation and the generalizability of study findings for these children is unclear [23]. While the possibility for drawing causal inferences about the reasons for symptom reduction is limited in observational trials, they can provide valuable information regarding whether a treatment is likely to be effective under real world conditions, across different patient types and settings.

Because this was an observational study it has specific strengths and limitations. The pre-study medication history of the children evaluated in the study was varied, with the majority of children switching from other MPH formulations at a range of doses, and there was no baseline medication/dose stabilization phase. Furthermore, administration of MPH did not follow a standardized treatment protocol and treatment plans were individualized. Differences in symptomatology, previous medication and actual medication are therefore confounded and interpretation of treatment effects is complicated. Low internal validity is one of the major drawbacks of observational studies. However, this analysis identifies clusters of patients that can be observed in common medical practice. Information about a patient’s initial situation and ADHD treatment in real world settings, including treatment regimes and treatment responses, is valuable for clinical practice as well as research. For the practitioner, knowledge about the typical subgroups of patients in routine clinical practice may be useful for treatment individualization. Furthermore, such studies are useful to show the need for treatment optimization.

There were several limitations to this study. First, this was an observational study with no (placebo) control group and parents were not blinded to the study treatment or dose, which may have influenced parent-ratings of ADHD symptoms. The use of a placebo control group would have allowed us to disentangle actual perceived improvements from bias based on hope or expectations of the parents. Yet, teachers were not formally informed of the change of treatment, and although they could have been told by parents or children, most were not aware. Second, the results for the previously treated group can only be generalized to a population in which a switch to MR-MPH is planned due to suboptimal efficacy with prior medication. Third, in particular for teacher ratings of ADHD symptoms, there was a substantial proportion of missing data. For this analysis, an inclusive missing data handling strategy was used and all available data were taken into account. Even when the underlying assumptions of such strategies are not met, they are still considered to be an improvement over ad hoc procedures like listwise deletion [65]. Advantages of this study are the relatively long period of observation, the large sample size and the use of multiple informants.

## Conclusions

Together with those by Sonuga-Barke et al. [20] and Swanson et al. [22], this is one of the few studies that has investigated the presence of latent subgroups in patients with ADHD with varying treatment responses after medication with MPH. All three studies indicate that there is heterogeneity in treatment response. The total population of children with ADHD – either previously treated or treatment naïve – receiving modified-release MPH under routine care conditions is best conceived as a composite of distinct subpopulations with varying treatment responses.

The current analysis revealed four subgroups after switching medication. Distinctive features of subgroups could be identified. Age, MPH dose at study start and conduct problems were particularly useful to discriminate between clusters of patients. Older children and children with few conduct problems were more likely to be members of a patient cluster with few symptoms at study start. Children with a low MPH dose in the beginning had a higher chance of being in the patient cluster with a strong treatment response. These results were in line with expectations. More research is needed to replicate these findings and to explore additional predictors to achieve a more complete picture of the differentiating characteristics of responsiveness subgroups. The current analyses should assist in identifying children likely to achieve a favourable treatment course with MPH and, additionally, those who are in need of alternative treatment options.

## Abbreviations

ADHD: Attention deficit hyperactivity disorder; BIC: Bayesian information criterion; COMACS: Comparison of methylphenidates in the analog classroom setting; DISYPS: Diagnostic system for mental disorders in children and adolescents; DSM-IV-TR: Diagnostic and statistical manual of mental disorders, 4^th^ edition, text revision; FBB-ADHD: German ADHD symptom checklist (Fremdbeurteilungsbogen für Aufmerksamkeitsdefizit-Hyperaktivitätsstörung); FIML: Full-information maximum-likelihood method; HKD: Hyperkinetic disorder; ICD-10: International classification of diseases, version 10; MPH: Methylphenidate; MPHpre: Daily dose (mg) of MHP before switch to MR-MPH; MPHVisit 1: Daily dose (mg) of MR-MPH as recorded at Visit 1; MPHVisit 2: Daily dose (mg) of MR-MPH as recorded at Visit 2; MR-MPH: Modified-release methylphenidate; MTA: Multimodal treatment study of children with ADHD; OBSEER: Observation of safety and effectiveness of Equasym XL in routine care; OR: Odds ratio; SD: Standard deviation; SDQ: Strengths and difficulties questionnaire.

## Competing interests

Christopher Hautmann has received travel grants from Shire Pharmaceuticals Ltd.

Aribert Rothenberger has acted as a consultant or on advisory boards and/or as a speaker for Lilly, Shire Pharmaceuticals Ltd, Medice, Novartis and UCB. He has received research support from Shire Pharmaceuticals Ltd, the German Research Foundation and Schwaabe, and travel and educational grants from Shire Pharmaceuticals Ltd.

Manfred Döpfner has received research grants and/or acted as a consultant or on advisory boards for German Research Foundation, Lilly, UCB, Shire Pharmaceuticals Ltd, Medice and Vifor.

## Authors’ contributions

CH has performed the statistical analysis and has drafted the manuscript. AR and MD have contributed to the conception and design of the study, the analyses and interpretation of the data, and the drafting and revising of the manuscript. All authors have approved the manuscript for submission.

## Authors’ information

Christopher Hautmann is a clinical psychologist and psychotherapist. His main research interests involve externalizing disorders, treatment evaluation and the application of advanced statistical methods in clinical research. Aribert Rothenberger is Head and Chair of Child and Adolescent Psychiatry at University Medicine Göttingen, Germany. Manfred Döpfner is Professor in Child and Adolescent Psychiatry and Head of the School of Child and Adolescent Psychotherapy at the University Hospital, Cologne, Germany.

## Pre-publication history

The pre-publication history for this paper can be accessed here:

http://www.biomedcentral.com/1471-244X/13/219/prepub
